# Relationships between stress indicators and sexual development during onset of maturity in broiler breeder roosters

**DOI:** 10.1016/j.psj.2025.106278

**Published:** 2025-12-15

**Authors:** J.M. Caliva, A. Marozzi, M.C. Nobili, H.A. Guidobaldi, R.H. Marin, J.M. Kembro

**Affiliations:** a Universidad Nacional de Córdoba (UNC), Facultad de Ciencias Exactas, Físicas y Naturales, Instituto de Ciencia y Tecnología de los Alimentos (ICTA), Córdoba, Argentina; b Consejo Nacional de Investigaciones Científicas y Técnicas (CONICET), Instituto de Investigaciones Biológicas y Tecnológicas (IIByT, CONICET-UNC), Córdoba, Argentina; c Instituto Académico Pedagógico de Ciencias Básicas y Aplicadas de la Universidad Nacional Villa María (IAPCByA-UNVM), Villa María, Córdoba, Argentina

**Keywords:** Rooster, Broiler breeders, Immune response, Reproduction, Sexual maturity

## Abstract

The onset of sexual maturity involves extensive physiological and metabolic changes modulated by internal and external cues. To ensure reproductive efficiency, broiler breeder management relies on strict feed programs to prevent overweight. However, this strategy can induce chronic stress in susceptible individuals, potentially affecting the immune system (i.e., increased heterophil/lymphocyte (H/L) ratio), compromising both welfare and sexual development. At the same time, sexual maturity can lead to increased energy allocation, which itself may modulate immune parameters. In this context, we evaluated the network of relationships among welfare and morphophysiological variables (body weight, breast condition, health status, H/L ratio, plasma testosterone levels, and testicular width) in 29-week-old Cobb roosters under commercial broiler breeder farm conditions. Our aim was to improve the understanding of inter-individual variability during the onset of sexual maturation, with a focus on the H/L ratio as a classical indicator of chronic distress. Sixty roosters were assessed, all showing optimal plumage and no signs of pododermatitis or hock burns, indicating good health and general welfare. Histograms revealed high variability in plasma testosterone levels, with values ranging from 0 to 4 ng/mL. (Cobb Vantress, 2022). Noticeably, around 33 % of the birds showed relatively low testosterone concentrations (below 0.46 ng/mL) and small testes (width < 1.84 mm), consistent with sexual immaturity. As expected, strong positive correlations were found between body weight and breast conformation. Interestingly, a moderate but significant (ρ=0.57; *P* < 0.05) correlation emerged between H/L ratio and testicular width. To further investigate these relationships, roosters were categorized by high (>0.95), intermediate (0.54–0.90), and low (<0.51) H/L ratio groups. Roosters with high H/L ratios were heavier with higher testosterone levels than those with low ratios. Overall, these findings suggest that the onset of male sexual maturity in broiler breeders is not merely a period of distress but also a stage of adaptive physiological adjustments, where H/L ratio may serve as a key marker linking stress responsiveness to reproductive activation.

## Introduction

The onset of sexual maturity involves extensive physiological and metabolic changes modulated by internal and external cues; in roosters, this includes an increase in plasma testosterone levels and testicular activity (Vizcarra et al., 2010). During this key stage of development, broiler breeders in commercial farms are subject to strict management protocols designed to maximize egg production and fertility (Cobb Vantress, 2022). These protocols involve lighting schedules aimed at synchronizing the onset of egg laying and reproductive behavior, as well as feed restriction to prevent breeders from becoming overweight. Because broilers are highly selected for rapid weight gain and efficient feed conversion, weight management in breeders requires particular attention. If roosters gain excessive weight (e.g., over 4.4 kg) and/or develop overly large breast conformations (>3), mating efficiency may decrease (Cobb Vantress, 2022). Conversely, a loss in body weight of more than 100 g can reduce sperm quality (Cobb Vantress, 2022). However, such feed restriction, while preventing overweight, could induce chronic stress in susceptible individuals, potentially compromising both welfare and sexual development.

Since chronic stress is linked to an individual’s perception of a challenge, inter-individual variability within a population is expected (Blas and Fairhurst, 2022). The physiological response to a stressor is characterized by prolonged adrenal activity, including increased corticosterone concentration, which alters white blood cell synthesis, decreasing lymphocyte and increasing heterophil counts (Davison et al., 1983). Consequently, the heterophil/lymphocyte (H/L) ratio is frequently used as an indicator of welfare quality and/or adverse conditions (“distress”) (Gross and Siegel, 1983). However, it is important to consider that adrenal activity is also modulated by metabolism, as evidenced by a recent meta-analysis showing that metabolic rate is an important driver of intra- and inter-individual glucocorticoid variations (Jimeno and Verhulst, 2023). Adrenal activity is also associated with the concept of allostasis, i.e. the process of achieving stability through change (Blas and Fairhurst, 2022). In this framework, the relationships between energy requirement at a given moment and the level of a specific allostasis mediators (including but not limited to glucocorticoids) interact in complex physiological ways to meet allostatic demands (Blas and Fairhurst, 2022). Hence, immune markers such as the H/L ratio not only can reflect conditions of poor welfare or chronic stress, but also predictable life-history transitions, including reproduction, aging, or sexual maturation, due to underlying resource-allocation trade-offs among physiological systems.

At 29 weeks of age, Cobb guidelines stipulate that up to 90 % of a broiler breeder flock can potentially reach sexual maturity (Cobb Vantress, 2022). However, in modern commercial settings, this is rarely evaluated directly, as farmers generally monitor more accessible variables such as body weight, breast condition, and overall fertility. The aim of this study was to gain insight into inter-individual variability during the onset of sexual maturation, focusing on the relevance of the H/L ratio as a classical distress indicator. We evaluated the network of relationships among welfare and morphophysiological variables—body weight, breast condition, health status, H/L ratio, plasma testosterone levels, and testicular width—in 29-week-old Cobb roosters in a commercial broiler breeder farm. Given the limited sample size, these findings are intended to motivate further investigation into broiler breeder physiology and behavior within commercial settings.

## Materials and methods

This study was conducted at a commercial broiler breeder farm, of the Cobb line, in central Argentina. All the procedures were approved by the Institutional Committee for the Use of Laboratory Animals (Acta 023). Animals were raised according to the standardized process of the Cobb breeders manual (Cobb Vantress, 2022). Briefly, after hatching, males and females were raised separated until one month of life. Then, animals were rehoused in mixed groups of males and females maintaining a proportion of one male for every nine females. Males (31 days of age) were divided according to size (based mainly on live weight) in three groups: heavy (more than 4 kg), intermediate (4 kgs), and light (less than 3.9 kg), and were housed in different barns accordingly to control feeding regime. Stocking density was 6 birds/m^2^. Barns were floor-tunneled with pad cooling, size 12 m x 150 m, i.e., 1800 m^2^. Nesting systems consist of 1 nest for 4 hens. The nest boxes (25 cm x long, 30 cm x high and 25 cm deep) had dry bedding and provided environmental comfort for females. The maximum height to jump to the perches to get into the nest was around 45 cm. Egg collection was done frequently to prevent having more than 3 eggs in each nest to avoid pre-incubation and broken eggs (Cobb Vantress, 2022). Males were fed once a day (at 4:00 am) with a feeder different from the one used to females, to avoid hens use, with water provided ad libitum. A photoperiod of 16 hours of light and 8 hours of darkness was maintained. In each house, two pens of 14 × 10 m (width x length, respectively) were created to facilitate the sampling process and assess welfare conditions on each individual.

For this study, to enhance variability we worked with two houses, corresponding to those containing individuals classified as heavy or lean upon housing. Blood samples were taken from the brachial vein of 30 males from each house immediately after capture, in a time not exceeding 2 minutes by bird. Samples were taken during two consecutive days for two hours, between 9:00 and 11:00 am, one day for each house. This brief sampling interval and standardized time window were used to minimize potential acute-handling and time-of-day effects on H/L ratio and testosterone measurements. A drop of each blood sample was immediately used to prepare direct smears for estimating the heterophil/lymphocyte (H/L) ratio, while the rest of the sample (1 mL) was centrifuged and stored in an Eppendorf tube for testosterone determinations. After blood extractions, males were identified by wing bands and released inside one of the pens, with females, maintaining the commercial density. Once all the extractions were completed, each male was weighed, the sanitary condition, welfare status, and morphometric variables were measured.

### Morphometrics variables

1. Body weight: Each individual was weighed using an electronic scale.
2. Breast width: Distance between the two shoulder joints, measured with a caliper. This measurement serves as a physical indicator of pectoral muscle development in birds.
3. Breast conformation (fleshing): scored on a 1–5 scale (1 = under-conditioned, very thin with no wing resistance; 5 = very wide breast with pronounced keel dimple; Cobb Vantress, 2022).
4. Comb area: calculated as length x ridge height, measured with a digital caliper.

### Blood and testicular measures

1. Heterophil/lymphocyte (H/L) ratio: Blood smears stained with May-Grünwald-Giemsa. About 100 leukocytes were counted (heterophils, lymphocytes, monocytes, basophils, eosinophils), and the H/L ratio calculated as heterophils/lymphocytes.
2. Testosterone blood levels (ng/mL): Aliquots (in duplicate) were analyzed by electrochemiluminescence (Elecsys Testosterone II - Cobas®). Manufacturer cross-reactivities were Androstendione: 2.66 %; Cortisol: 0.016 %; Cortisone: 0.002 %; Dexamethasone: 0.0004 %; Estradiol: 0.148 %; Estrone: non-detectable; 19-Norethisterone: 3.44 %; Prednisone: 0.016 %; Progesterone: 0.023 %, among others. For more details see https://elabdoc-prod.roche.com/eLD/api/downloads/8a44bb64-f477-ee11-2291-005056a71a5d?countryIsoCode=XG
3. Testicular width: assessed by ultrasound (Thielebein and Kozlowski, 2010). It was assessed using a portable ultrasound system (SonoScape X-V) equipped with a Micro-convex transducer operating at 5.4-10 MHz. Due to the high cloacal gas content, only testicular width was measured. This measurement was not possible in all males; however, measurements were successfully achieved in 86 % of the birds.

### Welfare indicators (adapted from the Welfare Quality® protocol)

1. Pectoral dermatitis 4-point scale, where zero means no lesion and three means severe lesion).
2. Abdominal injury 4-point scale, where zero means no injury and three means severe injury.
3. Clean plumage 4-point scale, where zero means clean plumage and three means very dirty plumage.
4. Absence of plumage 4-point scale, where zero means intact plumage with no visible dermis in the pectoral area and three means pectoral area without feather cover but without dermatitis.
5. Pododermatitis 5-point scale, where zero means no lesion and four means severe lesion.
6. Hock lesion 3-point scale, where zero means no lesion and two means severe lesion.

### Data and statistical analysis

All statistical tests were performed with RStudio. A Spearman correlation analysis was run to determine the degree of association between the variables. A Bonferroni-Holm Correction for Multiple Comparisons was used to adjust p-values using the MATLAB code *bonf_holm*. Network representation was used to visualize the level of correlation between variables, with the nodes representing variables and the color and width of the edges indicating the corresponding Spearman's rank correlation coefficient.

Plots were rendered in Matlab, using the functions *corrplot*, and *graph*. Dataset and code are available upon request.

To facilitate comparison of divergent H/L responses, roosters were categorized into low (H/L < 0.51), intermediate (H/L = 0.54–0.90), and high (H/L > 0.95) H/L ratio groups based on the distribution of observed values. These thresholds correspond to the lower 25 %, middle 50 %, and upper 25 % of the distribution, respectively and are consistent with previous studies proposing H/L threshold values (Gross and Siegel, 1983). To determine differences between categories, generalized linear mixed models (GLMM) were used, fixed factors were categories (Wicaksono et al., 2020), with house as a random effect. Based on the distribution of the response variables, the gamma family was used for weight, comb area, breast condition, breast width, blood testosterone levels, H/L ratio and testicular width. All analyses were performed with the packages Ime4 and glmmTMB and effect size was estimated using odds-ratio, in RStudio (R Core Team, 2022).

## Results and discussion

Animals presented scores between 0 and 1 in all welfare variables measured, suggesting that all males sampled were in good health.

**Fig. 1:**
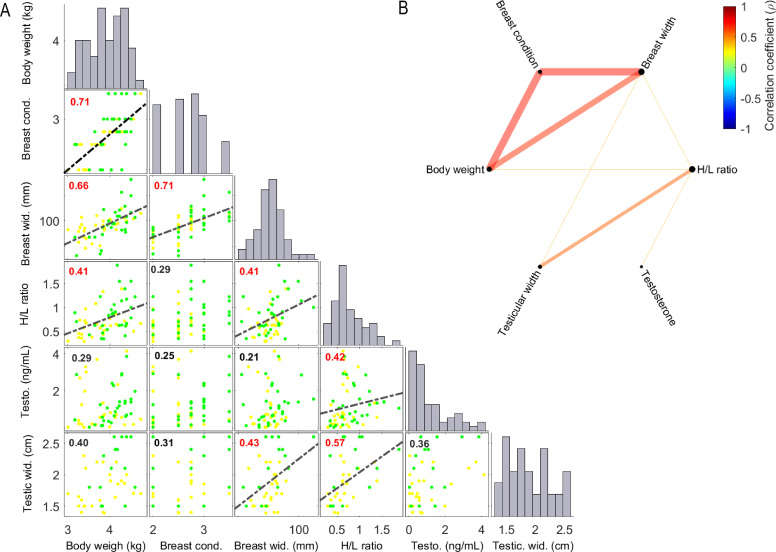
Relationships between morphometric, blood, and testicular measures in male broiler breeders. (A) The diagonal panels display histograms (grey vertical bars) representing the frequency distribution of each variable analyzed. Scatterplots show pairwise correlations between variables, with data points represented as circles. Circle color (yellow and green) indicates the barn studied. The correlation coefficient (ρ) is shown in the upper left corner of each plot. When correlations are significant (P < 0.05), the corresponding regression lines are displayed (black dashed lines). Heterophil and lymphocyte values as well as variables without significant correlations with others were omitted to facilitate visualization. From top to bottom: body weight (kg), breast condition, breast width (mm), heterophil/lymphocyte ratio, testosterone (ng/mL), and testicular width (cm). (B) Network representation of the same correlations shown in panel A, with line colors indicating the strength and direction of the correlation coefficient (ρ). Only statistically significant correlations are displayed (p < 0.05).

Fig. 1A shows both histograms of the frequency distribution and correlations among morphophysiological variables. The first histogram shows that the mean weight falls within the expected range (3.6 and 4.4 kg), with a coefficient of variation of 11 %, which is slightly higher than the recommended 8 −10 % (Cobb Vantress, 2022). These findings suggest that even in highly selected lines, individuals respond differentially to breeding environments, still showing an evident variability in flock uniformity. Additionally, 15 % of individuals (Fig. 1) exceeded the threshold of 4.4 kg, a weight associated with optimal reproductive performance (Cobb Vantress, 2022) and most roosters (67.8 %), showed breast condition scores between 2.5 and 3 (Fig. 1A.) which are considered optimal for breeding males (Cobb Vantress, 2022).

Plasma testosterone levels ranged from 0 to 4 ng/mL, with around 33 % of the birds showing relatively low concentrations (below 0.46 ng/mL) and small testes (< 1.84 mm) (Fig. 1A). These results suggest a delayed sexual maturity in a proportion of males, consistent with previous reports (Vizcarra et al., 2010). However, these values contrast with Cobb guidelines, which states that more than 90 % of the flock is expected to be reproductively active at 29 weeks of age (Cobb Vantress, 2022). Inter-individual variability in maturation may have consequences for fertility, hatchability, and semen quality, both in the short and long term (Hocking and Bernard, 2000; Behnamifar et al., 2025).

Histograms also showed that most roosters had H/L ratios around 0.7, with some individuals presenting values above 1 (Fig. 1A). Traditionally, such higher values have been associated with chronic stress in poultry (Gross and Siegel, 1983). The correlations between variables are also depicted in Fig. 1A, as well as represented as a network in Fig. 1B. As expected, a positive correlation was found between body weight and breast condition (rho = 0.71). Because all roosters weighed within the adult range, the observed interindividual variations could reflect differences in the impact of the feeding restriction program among birds rather than abnormal growth. As stated previously, males were only fed once a day and this restriction could lead to competition between animals when accessing food. If so, it would be expected that lean males are the ones more affected by this daily restriction and therefore, they would show signs of chronic stress response. In contrast, H/L values showed a positive (although weak; rho=0.41; Fig. 1A) correlation with breast area and body weight suggesting that lean birds, were not systematically showing a chronic stress response.

Moreover, H/L ratio correlated positively with both testicular width and testosterone, although the strength of these associations were intermediate (e.g., ρ ∼ 0.42-0.57; Fig. 1A,B). To further examine these relationships, roosters were classified according to quartiles into low (<0.51), intermediate (0.54-0.90), and high (>0.95) H/L groups. Body weight, breast conformation, testicular width and testosterone were all significantly lower in the low H/L group compared with the high H/L group (Table 1). These patterns suggest a potential link between the H/L ratio and the progression of reproductive maturation. Noteworthy, these results should be interpreted as associations suggestive of possible links between H/L ratio and reproductive maturation, rather than evidence of a direct or causal relationship.

**Table 1:** Morphometric, blood and testicular measures in male broiler breeders classified according to their heterophil/lymphocyte (H/L) ratio.

					Odds ratio	
	High H/L (Q4; n = 15)	Medium H/L (Q2,Q3; n = 30)	Low H/L (Q1; n = 15)	P-value	Medium vs High H/L	Low vs High H/L
Body weight (kg)	4.15±0.09^a^	3.95±0.08^a^	3.65±0.11^b^	0.02	0.95	0.88
Comb area (mm^2^)	4625±227^a^	4904±226^a^	4549^a^±148^a^	0.42	-	-
Breast condition	2.7 ± 0.1^a^	2.7 ± 0.08^a^	2.45±0.1^a^	0.20	-	-
Breast width (mm)	83.0 ± 3.9^a^	75.5 ± 1.5^b^	73.0 ± 2.2^b^	0.03	0.90	0.87
Testosterone (ng/mL)	1.3 ± 0.2^a^	1.5 ± 0.2^a^	0.4 ± 0.1^b^	0.0001	1.10	0.30
Heterophils (%)	41.9 ± 1.4^a^	31.6 ± 0.73^b^	23.1 ± 1.1^c^	0.0001	0.75	0.55
Lymphocytes (%)	34.9 ± 1.0^a^	45.5 0.98^b^	57.3 ± 1.7^c^	0.0001	1.64	1.30
Monocytes(%)	18.3 ± 1.4^a^	15.8 ± 1.07^a^	15.2 ± 2.0^a^	0.39	0.83	0.86
H/L ratio	1.26±0.07^a^	0.70±0.02^b^	0.40±0.02^c^	0.0001	0.91	0.52
Testicular width (cm)	2.16±0.09^a^	1.99±0.09^a^	1.60±0.06^b^	0.0001	0.92	0.74

Q1-Q4: quartiles estimated from the probability distribution of H/L.

a–c Mean ± standard error with different superscripts within a column are different at P < 0.05. Odds ratio was estimated when statistically significant between medium or low H/L groups versus the High H/L group.

One possible explanation could involve behavioral factors such as male-male competition for access to females. However, given the high female- to-male ratio (9:1), competition is unlikely to fully explain the observed associations (Cobb Vantress, 2022). Nevertheless, we cannot rule out the influence of unobserved social dynamics, as no behavioral measurements were collected. Another possibility is that physiological changes associated with the onset of sexual maturity may indirectly influence the H/L ratio, for example through shifts in energy allocation or endocrine adjustments (England et al., 2023) that can trigger adrenal hyperactivity and, consequently, an increase in H/L ratio (Davison et al., 1983; Jimeno and Verhulst, 2023). Additionally, individual health variation (e.g., mild or subclinical immune activation) could also affect H/L ratios independently of reproductive status. However, because no clinical signs were detected during our assessments, this interpretation remains speculative.

Taken together, the present findings suggest that H/L ratio during the onset of sexual maturity in broiler breeder males should not be interpreted solely as a marker of chronic distress. Instead, it may also capture physiological processes associated with reproductive activation. This potential dual role highlights the need for caution when using H/L ratio as a welfare indicator in breeding contexts, as inter-individual variability may relate to differences in reproductive status that could ultimately influence fertility and performance. Further longitudinal studies across different commercial conditions will be valuable to further clarity these relationships and strengthen the interpretation of H/L dynamics in broiler breeder males.

## CRediT authorship contribution statement

**J.M. Caliva:** Writing – review & editing, Writing – original draft, Visualization, Methodology, Investigation, Formal analysis, Data curation, Conceptualization. **A. Marozzi:** Writing – review & editing, Writing – original draft. **M.C. Nobili:** Methodology, Data curation. **H.A. Guidobaldi:** Writing – review & editing, Methodology, Investigation. **R.H. Marin:** Writing – review & editing, Validation, Supervision, Project administration, Investigation, Funding acquisition, Conceptualization. **J.M. Kembro:** Writing – review & editing, Visualization, Validation, Resources, Project administration, Methodology, Investigation, Funding acquisition, Formal analysis, Data curation, Conceptualization.

## Disclosures

No potential conflict of interest was reported by the authors.
